# Cell Cycle Regulation by NF-YC in *Drosophila* Eye Imaginal Disc: Implications for Synchronization in the Non-Proliferative Region

**DOI:** 10.3390/ijms241512203

**Published:** 2023-07-30

**Authors:** Anthony Avellino, Chen-Huan Peng, Ming-Der Lin

**Affiliations:** 1 Department of Molecular Biology and Human Genetics, Tzu Chi University, 701 Zhongyang Rd., Sec. 3, Hualien 97004, Taiwan; anthonyavellino39@gmail.com; 2 Department of Orthopedics, Hualien Tzu Chi Hospital, Buddhist Tzu Chi Medical Foundation, 707 Zhongyang Rd., Sec. 3, Hualien 97002, Taiwan; peng0913@gmail.com; 3 School of Medicine, Tzu Chi University, 701 Zhongyang Rd., Sec. 3, Hualien 97004, Taiwan; 4 Institute of Medical Sciences, Tzu Chi University, 701 Zhongyang Rd., Sec. 3, Hualien 97004, Taiwan

**Keywords:** Nuclear Factor-YC, eyes absent, morphogenetic furrow, cell cycle synchronization

## Abstract

Cell cycle progression during development is meticulously coordinated with differentiation. This is particularly evident in the *Drosophila* 3rd instar eye imaginal disc, where the cell cycle is synchronized and arrests at the G1 phase in the non-proliferative region (NPR), setting the stage for photoreceptor cell differentiation. Here, we identify the transcription factor Nuclear Factor-YC (NF-YC) as a crucial player in this finely tuned progression, elucidating its specific role in the synchronized movement of the morphogenetic furrow. Depletion of *NF-YC* leads to extended expression of Cyclin A (CycA) and Cyclin B (CycB) from the FMW to the NPR. Notably, *NF-YC* knockdown resulted in decreased expression of Eyes absent (Eya) but did not affect Decapentaplegic (Dpp) and Hedgehog (Hh). Our findings highlight the role of NF-YC in restricting the expression of CycA and CycB in the NPR, thereby facilitating cell-cycle synchronization. Moreover, we identify the transcriptional cofactor Eya as a downstream target of NF-YC, revealing a new regulatory pathway in *Drosophila* eye development. This study expands our understanding of NF-YC’s role from cell cycle control to encompass developmental processes.

## 1. Introduction

**Figure 1:**
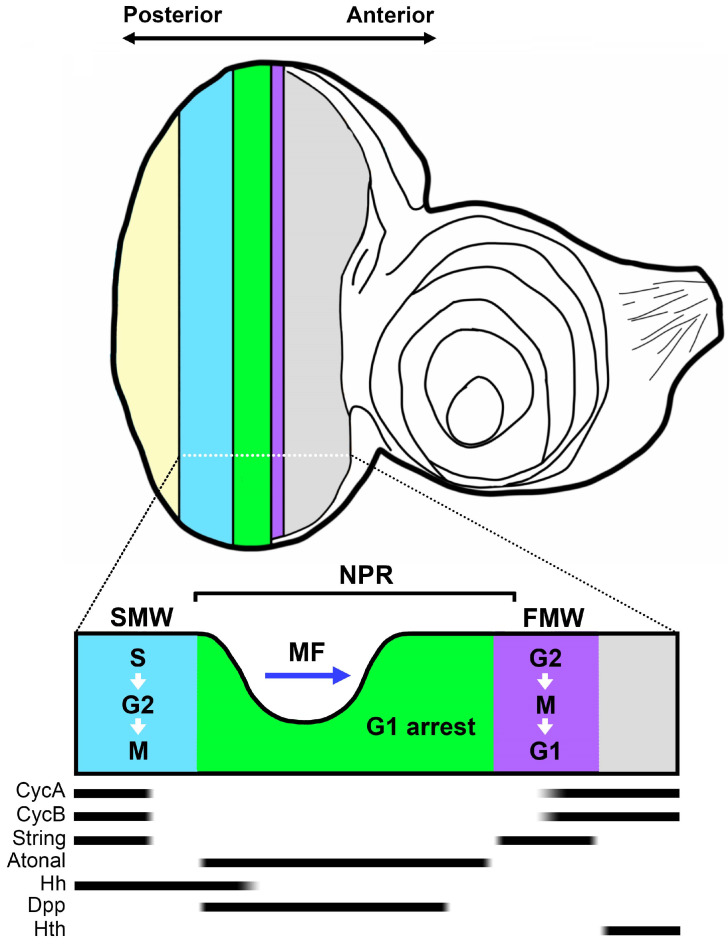
Illustration of cell cycle synchronization in the eye imaginal disc. In the anterior section of the eye disc (depicted in gray), cells undergo unsynchronized cell cycle progression. Prior to the non-proliferative region (NPR, highlighted in green), a significant band of String expression propels the cells into the M phase, forming the first mitotic wave (FMW, highlighted in purple) and subsequently entering the G1 phase in synchronization. In the NPR, the morphogenetic furrow (MF) moves anteriorly across the eye disc. The blue arrow indicates the direction of MF movement. Following the NPR, cells synchronously initiate the S phase and exit the M phase at different rates, creating the second mitotic wave (SMW, highlighted in blue). Atonal, expressed in the NPR, is mutually exclusive from Cyclin A (CycA) and Cyclin B (CycB) expression. CycA and CycB promote the S/G2 and G2/M phase transitions, respectively. Hedgehog (Hh) serves as a short-range signal, inducing Decapentaplegic (Dpp) expression within the NPR. Dpp functions to restrain Homothorax (Hth) expression in the unsynchronized region. The yellow color depicts the region containing differentiating photoreceptor cells. The black bars denote the expression range of molecular markers along the eye field.

Synchronization of the cell cycle is essential for shaping developmental processes. In this context, the eye imaginal disc of *Drosophila* 3rd instar larvae represents an exceptional in vivo model for studying cell cycle synchronization, as cell cycle progression within it is synchronized across specific cell columns (Figure 1). This disc, in the 3rd instar stage of *Drosophila*, features a groove-like structure termed the “morphogenetic furrow” (MF), which partitions the eye imaginal disc into an anterior undifferentiated region and a posterior differentiated region [1]. During larval development, the MF moves from the posterior to the anterior of the eye imaginal disc. The cells that differentiate behind the MF form clusters and ultimately transform into photoreceptor cells and accessory cells [2,3].

In tandem with a few columns of cells preceding it, the cells within the MF become arrested in the G1 phase of the cell cycle. This forms a non-proliferative region (NPR) in which the expression of Cyclin A (CycA), Cyclin B (CycB), Cyclin E (CycE), and *Drosophila* E2F transcription factor (dE2F) is suppressed [4,5,6,7,8]. In the NPR, the expression of the neurogenic gene *atonal* (*ato*) initiates the specification of neuronal cell fate [9]. Ahead of the G1 arrested region in the NPR, approximately 5–6 columns of cells are compelled to enter the M phase, constituting the “first mitotic wave” (FMW) [5]. The expression of String (Stg), a *Drosophila* Cdc25 homolog, propels these cells into mitosis within the FMW [10,11]. Ensuring the appropriate expression of Stg within the FMW necessitates the expression of retinal determination (RD) genes such as *twin of eyeless* (*toy*), *eyeless* (*ey*), *sine oculis* (*so*), and *eyes absent* (*eya*) [12]. Behind the NPR exists another mitotic region, known as the “second mitotic wave” (SMW), wherein an additional cell division cycle takes place. Given that some cells in the SMW do not immediately transition through the G2/M phase, the dividing cells within the SMW could encompass a span of approximately 20 cell diameters [5,12].

Decapentaplegic (Dpp) and Hedgehog (Hh) serve as the primary drivers of MF progression [13]. Dpp carries out signal transduction by binding to the heterodimeric receptor serine/threonine kinases, Thick veins (Tkv) and Punt (Put) [14,15,16,17]. Inhibition of Dpp signaling, as evidenced by *tkv* mutant clones at the posterior margin of the eye imaginal disc, results in a pronounced delay in MF progression [18]. Functioning upstream of Dpp in promoting MF progression, Hh’s depletion leads to the absence of Dpp expression in the MF [19]. Besides facilitating MF progression, Dpp and Hh signaling are both vital for maintaining G1 arrest in the NPR [4,20,21]. Dpp also plays an indispensable role in the formation of the FMW. Although Stg is necessary for promoting the G2/M transition at the FMW, its expression is negatively regulated by the homeodomain transcription factor Homothorax (Hth). To ensure appropriate *stg* expression at the FMW, the long-range effect of Dpp is necessary to suppress Hth expression in the FMW [22].

Nuclear Factor-Y (NF-Y) is a heterotrimeric transcription factor that binds to the CCAAT-box [23,24]. It consists of three subunits: NF-YA, NF-YB, and NF-YC. NF-YB and NF-YC form a heterodimer via their histone-fold motifs, providing a docking site for NF-YA, which contains a CCAAT box-DNA binding domain [25]. In mammalian proliferating cells, NF-Y can regulate the transcription of cell cycle regulators such as Cyclin A2, Cyclin B1/B2, Cdc2, Cdc25, and E2F1 [26,27,28,29,30,31], making it crucial for controlling the transition between S/G2 and G2/M phases. For instance, knocking down *NF-YC* in a colon cancer cell line impaired the G2/M phase transition [32]. Moreover, NF-Y is involved in DNA-damage-induced G2 arrest [31]. Unlike in proliferating cells, NF-Y is down-regulated in differentiating cells [33,34]. In *Drosophila* eye imaginal disc, NF-Y plays a role in differentiated cells. Knocking down *NF-YA* or *NF-YB* posterior to the MF impairs the differentiation of R7 photoreceptor cells [35,36]. In line with the effects of NF-YA and NF-YB on R7 photoreceptor cells, *NF-YC* mutants exhibit an axon targeting defect in R7 photoreceptor cells [37]. The role of NF-Y in the proliferating region of the eye field, however, remains unclear [35,36,38].

In this study, we investigate the function of NF-YC in regulating cell cycle synchronization in the NPR. Upon depletion of *NF-YC*, we observed ectopic expression of CycA and CycB in the NPR, indicating a G1 arrest defect. While the expression of Dpp, Hh, and Hth remained unaffected, the expression of Eya was reduced in the *NF-YC* knockdown clones. We further demonstrated that overexpression of Eya could rescue the phenotype induced by *NF-YC*-knockdown, in terms of ectopic expression of CycA and CycB in the NPR. These findings suggest that Eya acts downstream of NF-YC in the NPR to maintain cell-cycle synchronization during *Drosophila* eye development.

## 2. Results

### 2.1. Depletion of NF-YC Leads to Ectopic Expression of CycA and CycB in the Non-Proliferative Region

**Figure 2:**
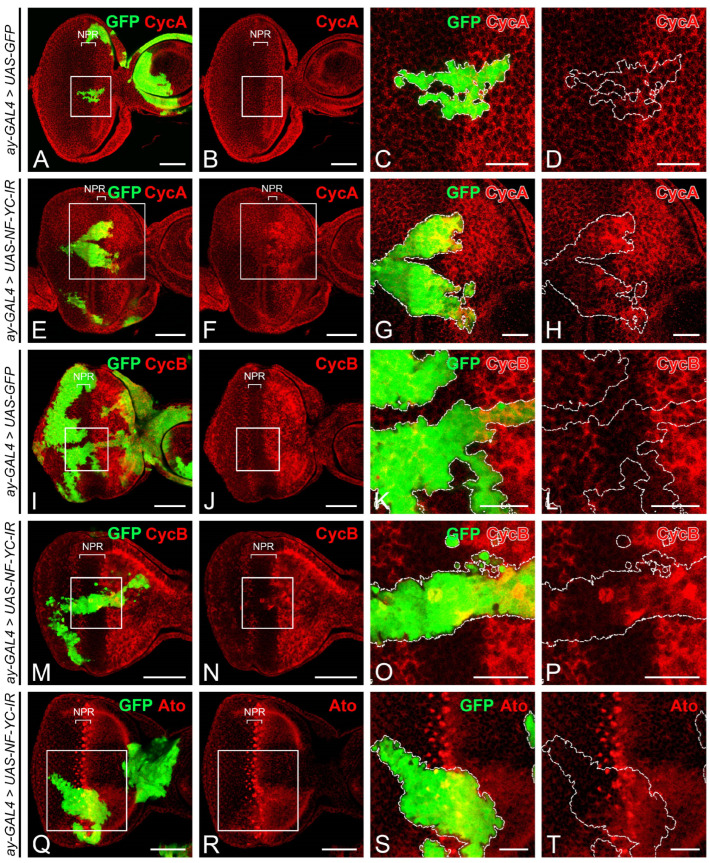
Depletion of *NF-YC* triggers ectopic expression of Cyclin A and Cyclin B and disrupts Atonal expression in the NPR. (**A**–**T**) Eye imaginal discs of 3rd instar larvae were immunostained with anti-Cyclin A (CycA) (red, (**A**–**H**)), anti-Cyclin B (CycB) (red, (**I**–**P**)), and anti-Atonal (Ato) antibodies (red, (**Q**–**T**)). GFP signal (green) identifies *NF-YC* RNAi flip-out clones. (**A**–**D**) Control eye disc with flip-out clones expressing only GFP. (**E**–**H**) Ectopic expression of CycA (red) is evident in *NF-YC*-knockdown clones (green) spanning the non-proliferative region (NPR). (**I**–**L**) Control eye disc with flip-out clones expressing only GFP. (**M**–**P**) *NF-YC*-knockdown clones (green) spanning the NPR show ectopic expression of CycB (red). (**Q**–**T**) Atonal (Ato) expression (red) is disrupted in *NF-YC*-knockdown clones (green) spanning the NPR. Genotypes in panels (**A**–**D**,**I**–**L**): *hsFLP*; *ay-GAL4*, *UAS-GFP*; Genotypes in panels E-H and M-T: *hsFLP/+*; *ay-GAL4, UAS-GFP/+*; *UAS-NF-YC-IR/+*. The white boxes in panels (**A**,**B**,**E**,**F**,**I**,**J**,**M**,**N**,**Q**,**R**) indicate the corresponding areas shown in panels (**C**,**D**,**G**,**H**,**K**,**L**,**O**,**P**,**S**,**T**), respectively. Dashed white lines delineate the boundaries of flip-out clones. Scale bars in panels (**A**,**B**,**E**,**F**,**I**,**J**,**M**,**N**,**Q**,**R**) represent 50 μm; panels (**C**,**D**,**G**,**H**,**K**,**L**,**O**,**P**,**S**,**T**) represent 20 μm. In all panels, anterior is to the right, dorsal is up.

To explore the role of NF-YC prior to photoreceptor cell differentiation, we generated GFP-marked *NF-YC* knockdown clones using the flip-out Gal4 technique [39]. In 3rd instar eye imaginal discs, cells within the non-proliferative region (NPR) ahead of the “morphogenetic furrow” (MF) are arrested in the G1 phase (Figure 1). In GFP control clones spanning the NPR, Cyclin A (CycA) (Figure 2A–D) and Cyclin B (CycB) (Figure 2I–L) were not expressed, consistent with the non-GFP control area in the NPR as previously reported [4,5]. In *NF-YC*-knockdown clones spanning the NPR, however, we observed ectopic expression of both CycA (Figure 2E–H) and CycB (Figure 2M–P). We found that in approximately 50% of *NF-YC*-knockdown clones spanning the NPR, the ectopic expression of CycA and CycB was restricted to the anterior part of the NPR, where cells are typically initiating G1 arrest [4]. Previous studies have shown that the proneural gene *atonal* (*ato*) is expressed only when cells exit the cell cycle and arrest at the G1 phase [22]. Correspondingly, we observed a disruption of Ato expression in the NPR when *NF-YC* was depleted (Figure 2Q–T). These results suggest a disruption of G1 arrest in the NPR when *NF-YC* knockdown clones span this region.

**Figure 3:**
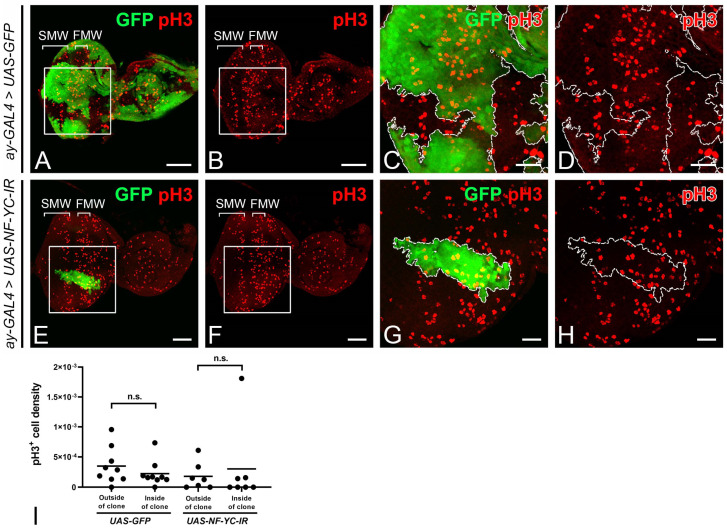
*NF-YC* knockdown does not induce ectopic mitotic activity in the NPR. (**A**–**H**) Eye imaginal discs of 3rd instar larvae were immunostained with anti-phospho-Histone 3 (pH3) antibody (red). GFP signal (green) denotes *NF-YC* RNAi flip-out clones. (**A**–**D**) Control eye disc with flip-out clones expressing only GFP. pH3 signals (red) are seen in the first and second mitotic waves (FMW and SMW) but absent in the NPR. (**E**–**H**) pH3 expression (red) remains unchanged in the NPR of *NF-YC*-knockdown clones (green). (**I**) Quantification of pH3-positive cells in the NPR reveals no significant alteration in cell density following *NF-YC* knockdown. Cell density is expressed as the number of pH3-positive cells per thousand pixels. A Student’s *t*-test was used to calculate *p*-values. n.s.: not significant. Genotypes in panels (**A**–**D**): *hsFLP*; *ay-GAL4, UAS-GFP*; panels (**E**–**H**): *hsFLP/+*; *ay-GAL4, UAS-GFP/+*; *UAS-NF-YC-IR/+*. The white boxes in panels (**A**,**B**,**E**,**F**) indicate the corresponding areas shown in panels (**C**,**D**,**G**,**H**), respectively. Dashed white lines indicate the boundary of flip-out clones. Scale bars in panels (**A**,**B**,**E**,**F**): 50 μm; panels (**C**,**D**,**G**,**H**): 20 μm. In all panels, anterior is to the right, dorsal is up.

To examine whether *NF-YC* depletion could drive cell cycle progression toward mitosis in the NPR, we stained for the mitotic marker phospho-Histone H3 (pH3). In control discs with GFP-expressing clones, mitotic cells marked by pH3 were concentrated in the first and second mitotic waves (FMW and SMW, respectively), but not in the NPR [5] (Figure 3A–D). Similar to the control eye disc, we did not observe an increase in the pH3 signal in *NF-YC*-knockdown clones spanning the NPR (Figure 3E–H). Consequently, the ectopic expression of CycA and CycB in the NPR induced by *NF-YC*-knockdown could not sustain cell cycle progression in the NPR.

### 2.2. NF-YC Is Not Essential for Expression of Dpp, Hh, and Hth in the Late 3rd Instar Eye Imaginal Disc

**Figure 4:**
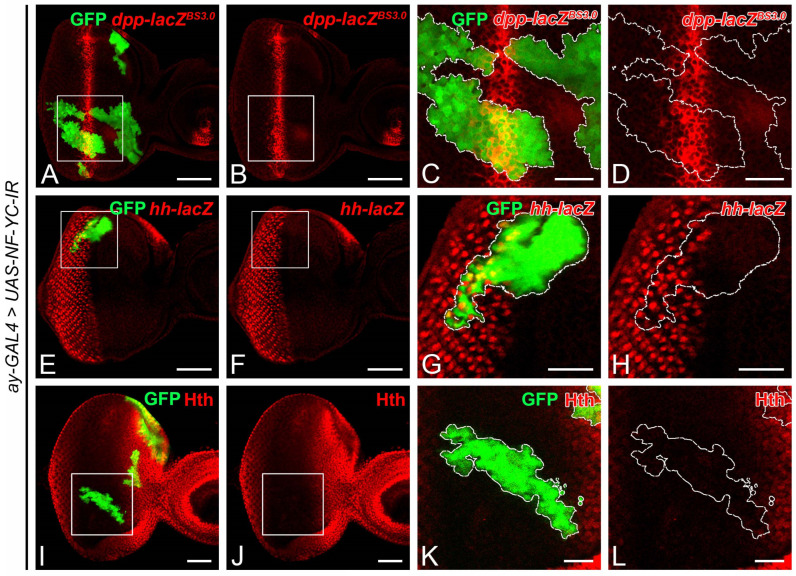
Depletion of *NF-YC* does not alter *decapentaplegic*, *hedgehog*, or Homothorax expression in 3rd instar eye imaginal discs. (**A**–**L**) Eye imaginal discs of 3rd instar larvae were immunostained with anti-β-galactosidase (protein product of *lacZ* reporter) (red, **A**–**H**) or anti-Homothorax (Hth) (red, (**I**–**L**)) antibodies. GFP signal (green) represents *NF-YC* RNAi flip-out clones. (**A**–**D**) Expression of *decapentaplegic* (*dpp*) reporter (*dpp-lacZ^BS3.0^*, red) remains unaffected in *NF-YC*-knockdown clones (green). Genotype: *hsFLP/+*; *ay-GAL4, UAS-GFP/dpp-lacZ^BS3.0^*; *UAS-NF-YC-IR/+*. (**E**–**H**) Expression of *hedgehog* (*hh*) reporter (*hh-lacZ*, red) is not affected by *NF-YC* knockdown (green). Genotype: *hsFLP/+*; *ay-GAL4, UAS-GFP/+*; *hh-lacZ, UAS-NF-YC-IR/+*. (**I**–**L**) Homothorax (Hth) protein expression (red) remains unchanged following *NF-YC* knockdown (green). Genotype: *hsFLP/+*; *ay-GAL4, UAS-GFP/+*; *UAS-NF-YC-IR/+*. The white boxes in panels (**A**,**B**,**E**,**F**,**I**,**J**) indicate the corresponding areas shown in panels (**C**,**D**,**G**,**H**,**K**,**L**), respectively. Dashed white lines indicate the boundary of flip-out clones. Scale bars in panels (**A**,**B**,**E**,**F**,**I**,**J**): 50 μm; panels (**C**,**D**,**G**,**H**,**K**,**L**): 20 μm. In all panels, anterior is to the right, dorsal is up.

Decapentaplegic (Dpp) and Hedgehog (Hh) have been reported to play pivotal roles in initiating G1 arrest in the NPR [4,7,21,40]. To evaluate whether *NF-YC* depletion could affect the expression of Dpp and Hh, we examined the expression of *dpp-lacZ* and *hh-lacZ* reporters in *NF-YC* knockdown clones. In the wild-type 3rd instar eye imaginal disc, dpp is expressed in the MF as a stripe [41], whereas Hh is expressed in the differentiating eye field, driving Dpp expression [19]. In *NF-YC*-knockdown clones covering the MF, we did not detect any change in *dpp-lacZ* expression (Figure 4A–D). Similarly, *hh-lacZ* expression was not altered in *NF-YC*-knockdown clones located posterior to the MF (Figure 4E–H). In addition to Dpp and Hh, ectopic expression of Homothorax (Hth) in the NPR also leads to extended CycB expression and loss of Ato expression [22]. Given that a disruption of Ato expression was observed in *NF-YC* knockdown clones (Figure 2Q–T), we further investigated whether *NF-YC* depletion could induce ectopic expression of Hth. In the 3rd instar eye disc with a MF progression, Hth is expressed anterior to the FMW but not in the FMW, NPR, and SMW [42]. In *NF-YC*-knockdown clones spanning the FMW and NPR, we did not detect ectopic expression of Hth (Figure 4I–L). Therefore, our findings suggest that NF-YC does not regulate the expression of Dpp, Hh, and Hth in the 3rd instar eye disc during MF progression.

### 2.3. NF-YC Depletion Reduces Expression of Transcriptional Cofactor Eyes Absent

**Figure 5:**
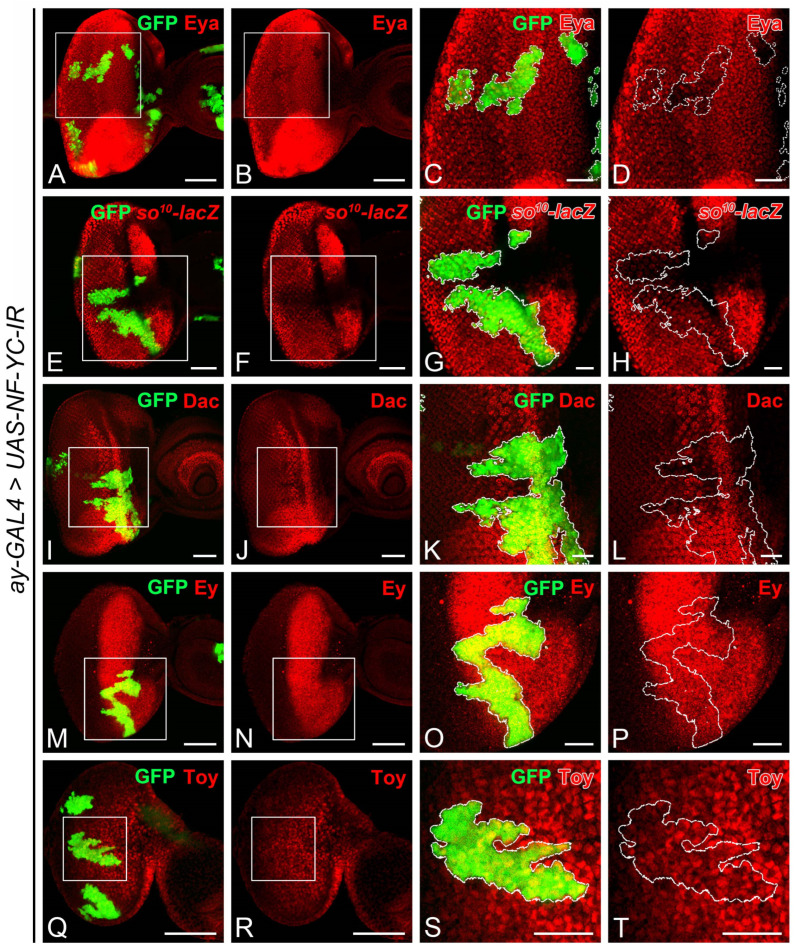
NF-YC is required for the expression of Eyes absent, but not Eyeless or Twin of eyeless in the developing eye disc. (**A**–**T**) Immunostaining of 3rd instar eye discs was performed using anti-Eyes absent (Eya) (red, (**A**–**D**)), anti-β-galactosidase (*lacZ* gene product) (red, (**E**–**H**)), anti-Dachshund (Dac) (red, (**I**–**L**)), anti-Eyeless (Ey) (red, (**M**–**P**)), and anti-Twin of eyeless (Toy) (red, (**Q**–**T**)) antibodies. The GFP signal (green) marks *NF-YC* RNAi flip-out clones. (**A**–**D**) Eya expression (red) was diminished in the *NF-YC*-knockdown clone (green). (**E**–**H**) The expression of the *so* reporter (*so^10^-lacZ*, red) remained largely unchanged in the *NF-YC*-knockdown clone (green). (**I**–**L**) Dac expression (red) was slightly reduced in the *NF-YC*-knockdown clone (green). (**M**–**T**) The expression of Ey (red, (**M**–**P**)) and Toy (red, (**Q**–**T**)) remained unaffected in the *NF-YC*-knockdown clone (green). Genotype in panels (**A**–**D**,**I**–**T**): *hsFLP/+*; *ay-GAL4, UAS-GFP/+*; *UAS-NF-YC-IR/+*; panels (**E**–**H**): *hsFLP/+*; *ay-GAL4, UAS-GFP/so^10^-lacZ*; *UAS-NF-YC-IR/+*. The white boxes in panels (**A**,**B**,**E**,**F**, **I**,**J**,**M**,**N**,**Q**,**R**) indicate the corresponding areas shown in panels (**C**,**D**,**G**,**H**,**K**,**L**,**O**,**P**,**S**,**T**), respectively. Dashed white lines denote the boundaries of flip-out clones. Scale bars in panels (**A**,**B**,**E**,**F**, **I**,**J**,**M**,**N**,**Q**,**R**): 50 μm; in panels (**C**,**D**,**G**,**H**,**K**,**L**,**O**,**P**,**S**,**T**): 20 μm. In all panels, anterior is to the right, dorsal is up.

The observed disruption of Ato expression in *NF-YC*-knockdown clones (Figure 2Q–T) mirrors the pattern seen when the expression of *eyes absent* (*eya*) is lost in the NPR [43]. Given that Eya acts as a transcriptional cofactor and physically interacts with Sine oculis (So) and Dachshund (Dac) [44,45,46], we speculated whether the expression of Eya, So, or Dac might be altered in *NF-YC*-knockdown clones. Notably, Eya expression diminished in *NF-YC*-knockdown clones spanning the NPR region (Figure 5A–D). While the expression level of the *so^10^-lacZ* reporter (Figure 5E–H) remained largely unchanged, Dac expression (Figure 5I–L) was reduced to a lesser extent in the *NF-YC*-knockdown clone. Given that Eya expression in the eye field can be regulated by Eyeless (Ey) and Twin of eyeless (Toy) [47,48], we further investigated whether Ey and Toy expressions would be impacted by NF-YC. Our results showed that their expressions remained unaltered in *NF-YC*-knockdown clones (Figure 5M–P for Ey, Figure 5Q–T for Toy).

**Figure 6:**
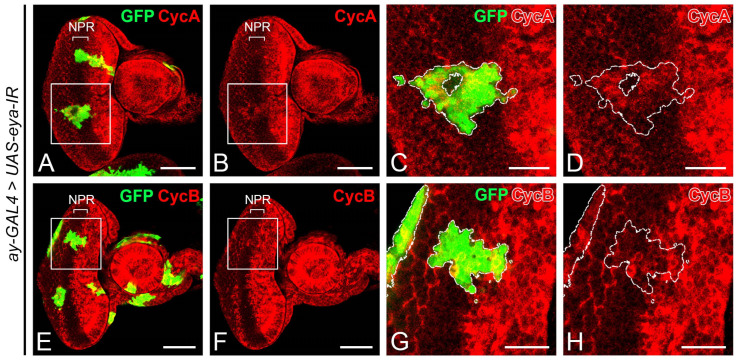
Ectopic expression of Cyclin A and Cyclin B is induced by the knockdown of *eya*. (**A**–**H**) Immunostaining of 3rd instar eye discs was performed with anti-Cyclin A (CycA) (red, (**A**–**D**)) or anti-Cyclin B (CycB) (red, (**E**–**H**)) antibodies. The GFP signal (green) denotes *eya*-knockdown clones. (**A**–**D**) Ectopic expression of CycA (red) is observed in *eya*-knockdown clones (green) covering the non-proliferative region (NPR). (**E**–**H**) *eya*-knockdown clones (green) across the NPR exhibit ectopic expression of CycB (red). Genotype in panels (**A**–**H**): *hsFLP/+*; *ay-GAL4, UAS-GFP/UAS-eya-IR*. The white boxes in panels (**A**,**B**,**E**,**F**) indicate the corresponding areas shown in panels (**C**,**D**,**G**,**H**), respectively. Dashed white lines outline the boundaries of flip-out clones. Scale bars in panels (**A**,**B**,**E**,**F**): 50 μm; panels (**C**,**D**,**G**,**H**): 20 μm. In all panels, anterior is to the right, dorsal is up.

These findings led us to hypothesize that the reduction of Eya might be behind the ectopic expression of CycA and CycB in the NPR when *NF-YC* was depleted. To examine this hypothesis, we explored whether the knockdown of *eya* could induce the ectopic expression of CycA and CycB in the NPR. As expected, we detected an ectopic expression of CycA (Figure 6A–D) and CycB (Figure 6E–G) in the NPR of the *eya*-knockdown clones.

### 2.4. Eya Overexpression Rescues CycA and CycB Mislocalization Resulting from NF-YC Depletion

**Figure 7:**
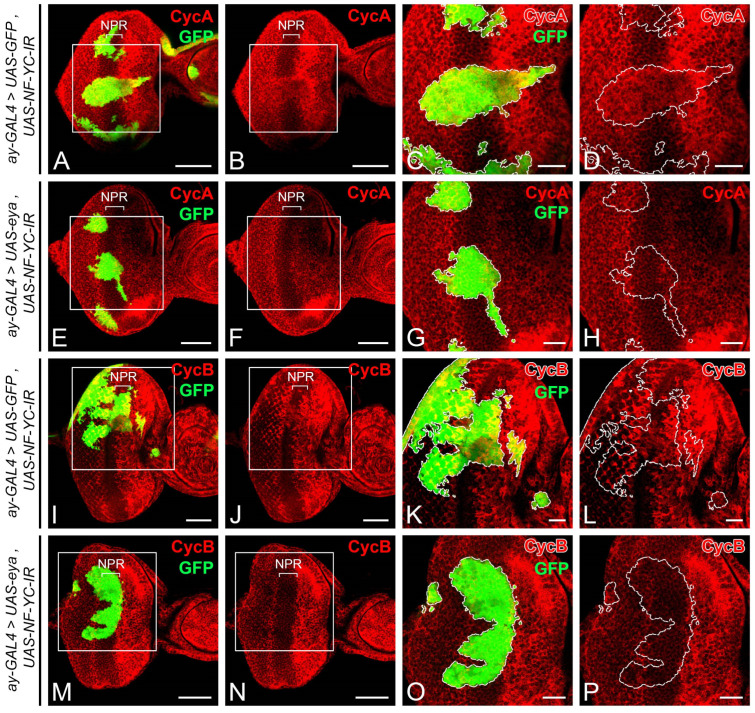
Eya overexpression rescues the ectopic expression of Cyclin A and Cyclin B in the NPR induced by *NF-YC* knockdown. (**A**–**P**) 3rd instar eye discs were immunostained with the anti-Cyclin A (CycA) (red, (**A**–**H**)) or anti-Cyclin B (CycB) (red, (**I**–**P**)) antibodies. The GFP signal (green) represents *NF-YC* RNAi flip-out clones generated. (**A**–**D**,**I**–**L**) Eye discs with *NF-YC* RNAi flip-out clones (green) expressing an extra copy of the GFP. CycA (red, (**A**–**D**)) and CycB (red, (**I**–**L**)) were ectopically expressed in the NPR of the *NF-YC*-knockdown clone (green). (**E**–**H**,**M**–**P**) The ectopic expression of CycA (red, (**E**–**H**)) or CycB (red, (**M**–**P**)) in the NPR was not detected in the *NF-YC* RNAi flip-out clone (green) co-expressing *eya*. Genotype in panels (**A**–**D**,**I**–**L**): *hsFLP/+*; *ay-GAL4, UAS-GFP/+*; *UAS-NF-YC-IR/UAS-GFP*; panels (**E**–**H**,**M**–**P**): *hsFLP/+*; *ay-GAL4, UAS-GFP/+*; *UAS-NF-YC-IR/UAS-eya*. The white boxes in panels (**A**,**B**,**E**,**F**,**I**,**J**,**M**,**N**) indicate the corresponding areas shown in panels (**C**,**D**,**G**,**H**,**K**,**L**,**O**,**P**) respectively. Dashed white lines outline the boundary of flip-out clones. Scale bars in panels (**A**,**B**,**E**,**F**,**I**,**J**,**M**,**N**): 50 μm; panels (**C**,**D**,**G**,**H**,**K**,**L**,**O**,**P**): 20 μm. In all panels, the anterior is to the right, the dorsal is up.

Considering that the aberrant expression of CycA and CycB in the NPR of *NF-YC* knockdown clones (Figure 2E–H,M–P) might stem from decreased Eya expression (Figure 6), we aimed to further substantiate this hypothesis. We tested if overexpressing Eya could reverse the aberrant CycA and CycB expression when *NF-YC* was depleted. We first introduced an additional copy of GFP into the *NF-YC*-knockdown clones as a control and analyzed the expression patterns of CycA and CycB. In these control *NF-YC*-knockdown clones, we observed aberrant expression of both CycA (Figure 7A–D) and CycB (Figure 7I–L) in the NPR when the clones spanned from the first mitotic wave (FMW) to the NPR. Interestingly, when Eya was overexpressed in the *NF-YC*-knockdown clones, the abnormal CycA (Figure 7E–H) and CycB (Figure 7M–P) expressions in the NPR was absent. These findings suggest that Eya operates downstream of NF-YC, playing a crucial role in maintaining the G1 phase arrest in the NPR.

## 3. Discussion

In this study, we present evidence demonstrating that NF-YC plays a crucial role in restricting Cyclin A (CycA) and Cyclin B (CycB) expression in the non-proliferative region (NPR). We observed that the ectopic expression of CycA and CycB in the NPR only occurs when *NF-YC*-knockdown clones span from the first mitotic wave (FMW) to the NPR. This suggests that the ectopic expression of CycA and CycB in the NPR of *NF-YC*-knockdown clones could be due to a delayed reduction of these cyclins. We also show that Eyes absent (Eya) mediates the ectopic expression of CycA and CycB induced by *NF-YC* depletion. Our research expanded the current understanding of NF-YC’s role in controlling cell cycle progression and contributes to the knowledge of the developmental process regulated by the transcriptional cofactor Eya.

The synchronized progression of the morphogenetic furrow (MF) in the *Drosophila* eye imaginal disc is critical for the coordinated differentiation of photoreceptor cells. In the *Drosophila* eye imaginal disc during the third instar larval stage, the cells anterior to the MF are propelled into the M phase, forming the FMW. Subsequently, they enter the NPR, where the cells arrest in the G1 phase, thereby achieving synchronization (Figure 1). This synchronized cell cycle progression is disrupted when *NF-YC* is depleted in the NPR, resulting in an ectopic accumulation of CycA and CycB (Figure 2). CycA regulates the transition from the S phase to the G2 phase, while CycB controls the transition from the G2 phase into the M phase. Overexpression or dysregulation of these cyclins may contribute to abnormal cell proliferation, potentially leading to oncogenesis or tumor development [49]. Despite this, it appears that *Drosophila* eye disc cells may have mechanisms in place to prevent uncontrolled cell proliferation in the NPR, even in the context of *NF-YC* depletion when high levels of CycA and CycB are accumulated. Interestingly, we did not observe ectopic pH3 signals in the NPR of *NF-YC* knockdown clones (Figure 3E–H), indicating that cell proliferation did not proceed. Instead, we detected the expression of the cleaved effector caspase, Death caspase-1 (Dcp1), in *NF-YC* knockdown clones, suggesting that apoptosis is triggered after *NF-YC* depletion (Figure S1). This observation aligns well with previous findings in *Drosophila* showing that knockdown of *NF-YB* induced apoptosis and that NF-Y positively regulates the expression of the anti-apoptotic protein Bcl-2 [36]. Therefore, during *Drosophila* eye disc development, NF-YC is not only required for cell cycle synchronization during MF progression, but also plays a critical role in ensuring cell survival.

A few genes in *Drosophila*, including those carrying specific mutant alleles, have been identified to influence cell synchronization during MF progression. For instance, the expression of *string* (*stg*), which encodes a Cdc25 homolog [50], is essential for driving G2 phase cells into mitosis and is consequently crucial for the formation of the FMW [22,51]. In *stg^hwy^* mutant eye discs, cells that are expected to be arrested in the G1 phase anterior to the MF accumulate CycA and CycB ectopically, alongside a loss of the NPR and rendering the FMW undetectable [51]. Although *NF-YC* knockdown similarly results in the accumulation of CycA and CycB in the NPR, we do not concur that *stg* is a downstream target of NF-YC. If *stg* were indeed a downstream target of NF-YC, we would anticipate a significant reduction of pH3 signals in the FMW upon *NF-YC* depletion. Contrary to this expectation, we detected no changes in pH3 signals in *NF-YC*-knockdown clones spanning the FMW (Figure 3E–I). Furthermore, previous studies suggest that *stg* transcription in the FMW is negatively regulated by Hth [12]. Yet, in *NF-YC*-knockdown clones spanning the FMW, we observed no corresponding changes in Hth expression (Figure 4I–L). These results lead us to propose that *stg* is not a downstream target of NF-YC in the regulation of cell cycle synchronization during MF progression. Roughex (Rux), a unique *Drosophila* inhibitor of G1 progression with no homologs in vertebrates, is also essential for G1 arrest in the NPR [5]. Mechanistically, Rux inhibits the accumulation of CycA in early G1 by targeting it for degradation [6,52]. In *rux* mutant eye discs, cells in the NPR accumulate CycA and CycB, evade G1 arrest, and prematurely enter the S and M phases [5,6]. There is a subtle difference between the *rux* mutant and the *NF-YC* knockdown clones in the NPR: while *rux* mutants push the cell into mitosis, knockdown of *NF-YC* does not (Figure 3E–H). Given these phenotypic differences, it seems improbable that the effects of *NF-YC* on CycA and CycB in the NPR is mediated through Rux. Decapentaplegic (Dpp) is another pivotal factor for maintaining G1 arrest in the NPR. When Dpp signaling is disrupted, as in the mutant clone of its receptor *thick veins* (*tkv*), the expression of CycA and CycB extends into the NPR from the FMW [20,53]. Due to these similar characteristics, we speculated that the regulation of NPR by *NF-YC* could be related to Dpp signaling. However, we found that *NF-YC* does not interfere with Dpp expression because we observed no decrease in *dpp* expression in *NF-YC*-knockdown clones (Figure 4A–D). Furthermore, NF-YC might not affect Dpp signal transduction. While we did not directly assess Dpp signaling in *NF-YC*-knockdown clones, inspecting Hth expression offers some insight. Given that Dpp signaling is essential for suppressing Hth expression anterior to the MF [42], our finding that *NF-YC* depletion could not induce ectopic Hth expression in the NPR or FMW (Figure 4I–L) suggests that Dpp signaling likely remains intact. While NF-YC may not be directly involved in the regulation of *dpp* expression or its signal transduction, we cannot discount the possibility that Dpp signaling might control the G1 arrest in NPR by influencing *NF-YC* expression. Nonetheless, this hypothesis requires further investigation.

We found that *NF-YC* knockdown led to decreased Eya expression (Figure 5A–D). In addition, *eya* depletion phenocopied the ectopic expression of CycA and CycB seen in *NF-YC*-knockdown clones (Figure 6). These results align with a previous study indicating that Eya reduction causes a delay in G1 arrest, demonstrated by CycB’s ectopic expression in the NPR [43]. Crucially, we were able to reverse the ectopic expression of CycA and CycB in *NF-YC*-knockdown clones through Eya overexpression (Figure 7E–H,M–P). These findings suggest that Eya operates downstream of NF-YC in the NPR to facilitate the transition from the M phase to G1 arrest. Alongside Eya, we also noticed a slight decrease in So and Dac expression when *NF-YC* was depleted (Figure 5E–L). In the early third instar eye disc, before MF formation, Dpp signaling is necessary for *eya* expression [54]. However, post MF initiation, *eya* expression becomes Dpp signaling-independent [54]. Consequently, *eya* expression in the NPR is not dictated by Dpp signaling. Instead, a positive feedback loop may exist to sustain *eya* expression. While the detailed mechanism for Eya regulation post MF formation remains elusive, prior studies have shown that Dac—though not So—can activate *eya*’s eye-specific enhancer when ectopically expressed in the antenna disc [45,55]. To date, no known protein factors besides Dac have been implicated in NPR *eya* regulation, positioning NF-YC as a potential novel regulator for *eya* expression post MF initiation. Yet, the specifics of this regulatory process need further exploration.

## 4. Materials and Methods

### 4.1. Drosophila Stocks and Genetics

*Drosophila* stocks were raised at 25 °C on a standard cornmeal medium. We obtained the following stocks from the Bloomington *Drosophila* Stock Center (BDSC), Kyoto Stock Center, or the Vienna *Drosophila* Resource Center (VDRC): *UAS-NF-YC-IR* (VDRC, stock No. 41034), *hs-FLP* (BDSC, stock No. 1929), *ay-GAL4*, *UAS-GFP* (BDSC, stock No. 4411), *hh-lacZ* (BDSC, stock No. 5530), *UAS-eya-IR* (VDRC, stock No. 108071), and *UAS-eya* (Kyoto Stock Center, stock No. 108356). Other *Drosophila* stocks used in this study include *dpp-lacZ^BS3.0^* [56] and *so^10^-lacZ* [57]. For flip-out clone generation, we collected eggs over a six-hour interval at 25 °C. We subjected first instar larvae to a two-hour heat shock at 37 °C. We manually dissected the eye-imaginal discs of wandering third instar larvae for whole-mount immunostaining.

### 4.2. Drosophila Whole-Mount Immunostaining

We dissected the eye imaginal disc from L3 larvae and subjected it to an immunostaining procedure as described by Lin et al., 2013 [58]. We obtained primary antibodies from the Developmental Studies Hybridoma Bank (DSHB) at the University of Iowa: mouse anti-CycA (DSHB, product ID: A12, 1:250 dilution), mouse anti-CycB (DSHB, product ID: F2F4, 1:1000 dilution), mouse anti-β-Galactosidase (DSHB, product ID: 40-1a, 1:200 dilution), mouse anti-Eya (DSHB, product ID: eya10H6, 1:1000 dilution), and mouse anti-Dac (DSHB, product ID: mAbdac1-1, 1:1000 dilution), mouse anti-Ey (DSHB, product ID: eyeless, 1:2000 dilution). We also used other primary antibodies in this study, including rabbit anti-pH3 (Cell Signaling Technology, Danvers, MA, USA, Cat. No. 9701, 1:1000 dilution), rabbit anti-cleaved *Drosophila* Dcp-1 (Cell Signaling Technology, Cat. No. 9578, 1:400 dilution), guinea pig anti-Toy (gift from Uwe Walldorf, 1:200 dilution), and rabbit anti-Hth (1:5000 dilution) [59]. The secondary antibodies used were goat anti-mouse IgG Alexa 568 Conjugated (Thermo Fisher Scientific Invitrogen, Waltham, MA, USA, Cat. No. A11004, 1:200 dilution), goat anti-guinea pig IgG Cy3 conjugated (Sigma-Aldrich, Burlington, MA, USA, Cat. No. AP108C, 1:200 dilution), and goat anti-rabbit IgG Alexa 568 Conjugated (Thermo Fisher Scientific Invitrogen, Cat. No. A11011, 1:200 dilution). For nuclear stain, DAPI solution (1 mg/mL) was used (Sigma-Aldrich, Cat. No. MBD0015, 1:1000 dilution). We captured and analyzed images using the Nikon A1+ Confocal microscope (Nikon Corporation, Tokyo, Japan) and Zeiss LSM 900 Confocal microscope (Zeiss, Oberkochen, Germany).

## Data Availability

Data are contained within the article.

## Supplementary Materials

The following supporting information can be downloaded at: https://www.mdpi.com/article/10.3390/ijms241512203/s1.
