# Supplementary material for: Cell Cycle Regulation by NF-YC in Drosophila Eye Imaginal Disc: Implications for Synchronization in the Non-Proliferative Region
Source: Int J Mol Sci. 2023 Jul 30;24(15):12203. doi: 10.3390/ijms241512203 (PMC10418845; doi:10.3390/ijms241512203)
Supplement: Supplementary file 1 [file ijms-24-12203-s001.zip › ijms-2511862-supplementary.pdf]

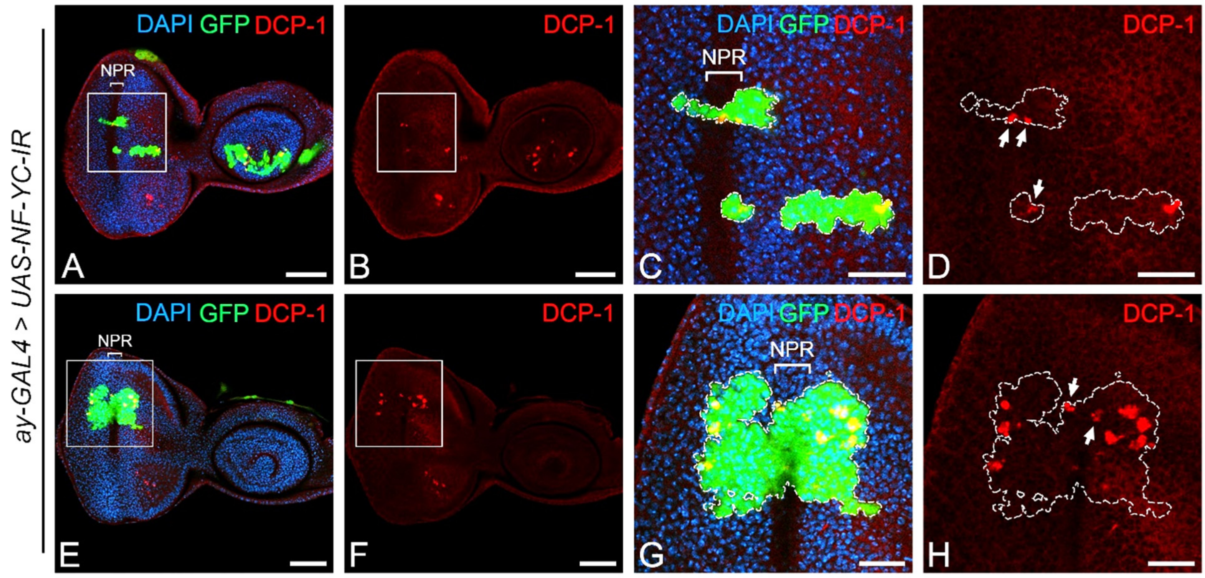

Figure S1. Depletion of *NF-YC* induces apoptosis. (A-H) Eye imaginal discs of 3rd instar larvae were immunostained with anti-cleaved *Drosophila* Death caspase-1 (DCP-1) antibody (red) to visualize the apoptotic cells. GFP signal (green) denotes *NF-YC* RNAi flip-out clones, and DAPI (blue) counterstains the nuclei. Within the *NF-YC*-knockdown clones (green), a clear DCP-1 signal (red) can be seen. The arrows in panels D and H point to apoptotic cells expressing DCP-1 (red) in the NPR. Genotype: *hsFLP/+; ay-GAL4, UAS-GFP/+; UAS-NF-YC-IR/+*. The white boxes in panels A, B, E, and F indicate the corresponding areas shown in panels C, D, G, and H, respectively. Dashed white lines delineate the boundaries of flip-out clones. Scale bars in panels A, B, E, and F: 50 µm; panels C, D, G, and H: 20 µm. In all panels, anterior is to the right, dorsal is up.
